# Imitation Is Not Just the Sincerest Form of Flattery—It Is the Sincerest Form of Learning: Reflecting on Rotation Mastopexy

**DOI:** 10.1093/asj/sjae063

**Published:** 2024-03-22

**Authors:** Niamh Corduff

I have read with interest a recent article highlighted in an Instagram post by *Aesthetic Surgery Journal*, titled “Inferior and Medially Based Parenchymal Rotation Flap: A New Mastopexy Technique for Replacing Breast Volume After Breast Implant Explantation” by Sözer and Sibar, which was published in the December 2023 issue of *Aesthetic Surgery Journal.*^1^

Upon reading the article, I noticed that the use of a parenchymal rotation flap to fill the upper pole was described in the paper as “new.” This, in fact, is not a recent innovation. In 2009, Corduff and Taylor published a study titled “Rotation Mastopexy: An Anatomical Approach” in *Aesthetic Plastic Surgery*, in which we described a superomedially based parenchymal rotation flap for mastopexy.^2^ Among the described series of 25 patients, 7 underwent bilateral removal of implants, specifically addressing the issue of filling the upper pole after breast implant removal. This technique was also published in Betsy Hall-Finlay's textbook on breast surgery techniques in 2010.^3^

The concept of a rotation flap to fill the deficit in the upper pole is well described in all these publications, however there are some differences in technique to note.

First, there are differences in the surgical planning, skin excision, and subsequent visible scarring. The original technique, described in 2009 by Corduff and Taylor, utilized a vertical closure with skin excision tailored at the end of the procedure. This approach avoids a potentially unsightly long inframammary scar extending laterally and medially associated with the Wise pattern.^4^ Moreover, it facilitates better “coning” and projection of the breast shape compared to the closure of a Wise pattern, which tends to flatten the new breast mound.^5^

Second, it is the author’s experience that there is no need to subdivide the parenchymal flap as described by Sözer and Sibar, in which a superior pedicle perfuses the nipple-areolar complex and an additional inferomedial flap is utilized for the rotation flap. An understanding of the functional anatomical circulation of the breast allows for 1 large parenchymal flap based on the second and third internal thoracic perforators. The blood supply of the breast follows a course from the major peripheral perforators, coursing centrally toward a subareolar plexus. From there, it continues downward into the breast gland.^6,7^ One large parenchymal flap, being superomedially based, can be fully freed inferomedially and laterally by dissecting between the capsule of the breast and subcutaneous tissue from a vertical elliptical incision approach and then lifted off the chest wall from an inferior and lateral approach.^7^ The capsule of the breast implant can be left attached to the undersurface of the flap or removed where indicated. This technique was applied exclusively by the author to manage the upper pole deficit after removal of breast implants in patients who did not want them replaced. In over 100 patients operated on from 2009 to the author’s retirement from operative practice in 2015 there were no cases of fat necrosis, reflecting the robust vascularization of the rotation flap.

As the field of mastopexy evolves, and with more patients seeking removal of breast implants, these advancements and refinements in surgical techniques are valuable. It is however important to accurately represent the historical context and evolution of these procedures. I believe that this clarification adds valuable context to the discussion surrounding mastopexy techniques and their variations. That said, I wish to congratulate the authors Sözer and Sibar for publishing their approach to the rotation mastopexy and reiterating its usefulness as more patients are seeking removal of their breast implants.
